# Natural Variants of the KPC-2 Carbapenemase have Evolved Increased Catalytic Efficiency for Ceftazidime Hydrolysis at the Cost of Enzyme Stability

**DOI:** 10.1371/journal.ppat.1004949

**Published:** 2015-06-01

**Authors:** Shrenik C. Mehta, Kacie Rice, Timothy Palzkill

**Affiliations:** Department of Pharmacology, Baylor College of Medicine, Houston, Texas, United States of America; Case Western Reserve University School of Medicine, UNITED STATES

## Abstract

The spread of β-lactamases that hydrolyze penicillins, cephalosporins and carbapenems among Gram-negative bacteria has limited options for treating bacterial infections. Initially, *Klebsiella pneumoniae* carbapenemase-2 (KPC-2) emerged as a widespread carbapenem hydrolyzing β-lactamase that also hydrolyzes penicillins and cephalosporins but not cephamycins and ceftazidime. In recent years, single and double amino acid substitution variants of KPC-2 have emerged among clinical isolates that show increased resistance to ceftazidime. Because it confers multi-drug resistance, KPC β-lactamase is a threat to public health. In this study, the evolution of KPC-2 function was determined in nine clinically isolated variants by examining the effects of the substitutions on enzyme kinetic parameters, protein stability and antibiotic resistance profile. The results indicate that the amino acid substitutions associated with KPC-2 natural variants lead to increased catalytic efficiency for ceftazidime hydrolysis and a consequent increase in ceftazidime resistance. Single substitutions lead to modest increases in catalytic activity while the double mutants exhibit significantly increased ceftazidime hydrolysis and resistance levels. The P104R, V240G and H274Y substitutions in single and double mutant combinations lead to the largest increases in ceftazidime hydrolysis and resistance. Molecular modeling suggests that the P104R and H274Y mutations could facilitate ceftazidime hydrolysis through increased hydrogen bonding interactions with the substrate while the V240G substitution may enhance backbone flexibility so that larger substrates might be accommodated in the active site. Additionally, we observed a strong correlation between gain of catalytic function for ceftazidime hydrolysis and loss of enzyme stability, which is in agreement with the ‘stability-function tradeoff’ phenomenon. The high T_m_ of KPC-2 (66.5°C) provides an evolutionary advantage as compared to other class A enzymes such as TEM (51.5°C) and CTX-M (51°C) in that it can acquire multiple destabilizing substitutions without losing the ability to fold into a functional enzyme.

## Author Summary

The absence of new antibiotics combined with the emergence of antibiotic-resistance enzymes like KPC-2 that can inactivate most β-lactam antibiotics has resulted in a longer duration of medical treatment, higher costs of medical care, and increased mortality. In recent years, a number of amino acid sequence variants of KPC-2 have been identified in clinical isolates worldwide suggesting continued evolution of resistance in KPC-2. In this study we have characterized nine clinically isolated variants of KPC-2 (KPC-3 to -11) that differ from the initial KPC-2 isolate by one to two amino acids. The KPC variants confer increased resistance to the antibiotic ceftazidime as compared to KPC-2. This increase in resistance is correlated with improved ability of the variant enzymes to hydrolyze the antibiotic. Additionally, the changes associated with increased ceftazidime hydrolysis also reduce the thermal stability of the enzyme, indicating the mutations that assist catalysis come with a cost on the overall stability of the enzyme. The high thermal stability of KPC-2 allows destabilizing mutations that enhance catalysis to accumulate while the enzyme retains a folded, functional structure. Thus, the high stability of KPC-2 provides an evolutionary advantage to acquire multiple mutations and retain function as compared to other β-lactamase enzymes.

## Introduction

Because of their broad-spectrum activity, safety and favorable pharmacokinetic properties [1], β-lactam antibiotics have been the drugs of choice to treat bacterial infections. While antibiotics have helped save millions of lives, the extensive use of these drugs has resulted in the emergence of antibiotic resistant bacterial strains. This problem is compounded by the ability of these organisms to acquire mutations or obtain genes encoding antibiotic-inactivating enzymes from other bacteria, thereby reducing the efficacy of drugs. Thus, treating antibiotic resistant bacterial infections is a complex clinical challenge [2].

**Fig 1:**
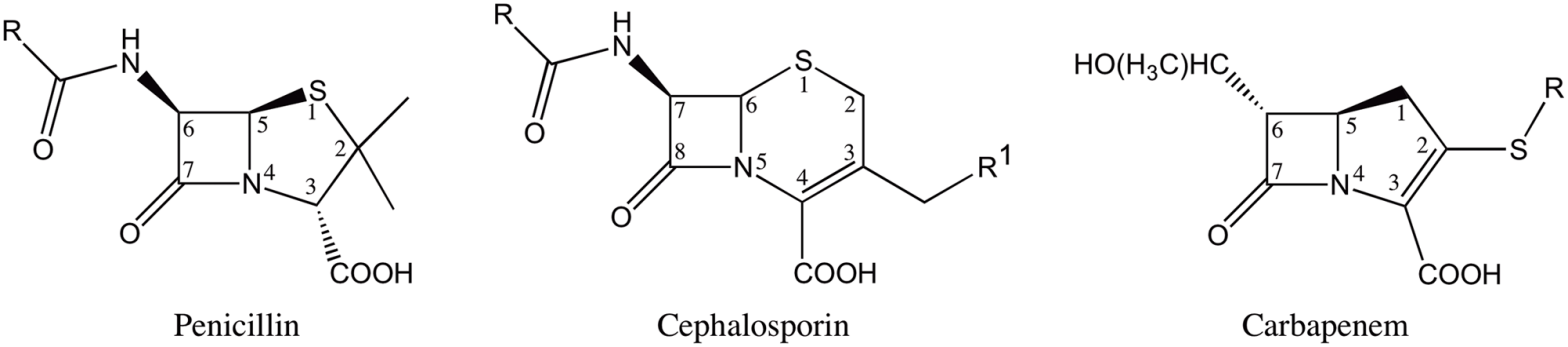
Structures of β-lactam antibiotics with positions numbered.

The β-lactam antibiotics contain a characteristic four-membered β-lactam ring and act as covalent inhibitors of the essential transpeptidase enzymes known as penicillin binding proteins (PBP’s). The β-lactam antibiotics are classified into different groups based on their chemical structure [3]. The most clinically relevant classes are penicillins, cephalosporins and carbapenems (Fig 1). The penicillins and cephalosporins contain the β-lactam ring fused to a five or six-membered ring, respectively. The carbapenems consist of the β-lactam ring fused to a five-membered ring with a carbon atom replacing the sulfur at the C-1 position along with an unsaturated C2-C3 bond [4] (Fig 1). The presence of a *6-α*-hydroxyethyl side-chain at the C-6 position of the β-lactam nucleus is a feature that distinguishes the carbapenems from the penicillins and cephalosporins that have a *6-β*- or *7-β*-acylamino side-chain in the same position, respectively [5] (Fig 1). In addition to being a structural distinguishing factor for the carbapenems, the *6-α-*hydroxyethyl side-chain is also responsible for the broad-spectrum activity of the carbapenem antibiotics [6–8].

Resistance to the β-lactam antibiotics is mediated largely through β-lactamase enzymes. These enzymes hydrolyze the β-lactam amide bond rendering the antibiotic inactive. The β-lactamases have been grouped into four classes based on the primary sequence homology [9]. Enzymes belonging to classes A, C and D catalyze β-lactam hydrolysis using a catalytic serine, while class B consists of metallo-enzymes that function using one or two zinc ions [10]. The carbapenem antibiotics are considered the last line of therapy as they are resistant to the action of most β-lactamases [4]. In fact, carbapenems directly inhibit some β-lactamases by forming a stable acyl-enzyme intermediate [6–8]. However, the increasing use of carbapenems has led to the emergence of carbapenem hydrolyzing β-lactamases [11]. Carbapenemase activity has been reported in class A, B and D β-lactamases [12]. In particular, resistance to carbapenems mediated by class A enzymes such as KPC-2, SME-1-3, IMI-1-2, SFC-1, NmcA, GES-2 and GES-4 to 6 poses a serious clinical threat [13]. Among these class A carbapenemases, KPC-2 is the most clinically important enzyme due to its prevalence in enteric bacteria [14]. Additionally, the presence of *bla*_KPC-2_ gene on the mobile transposon Tn*4401* has facilitated its dissemination among Gram-negative bacteria [15].

**Fig 2:**
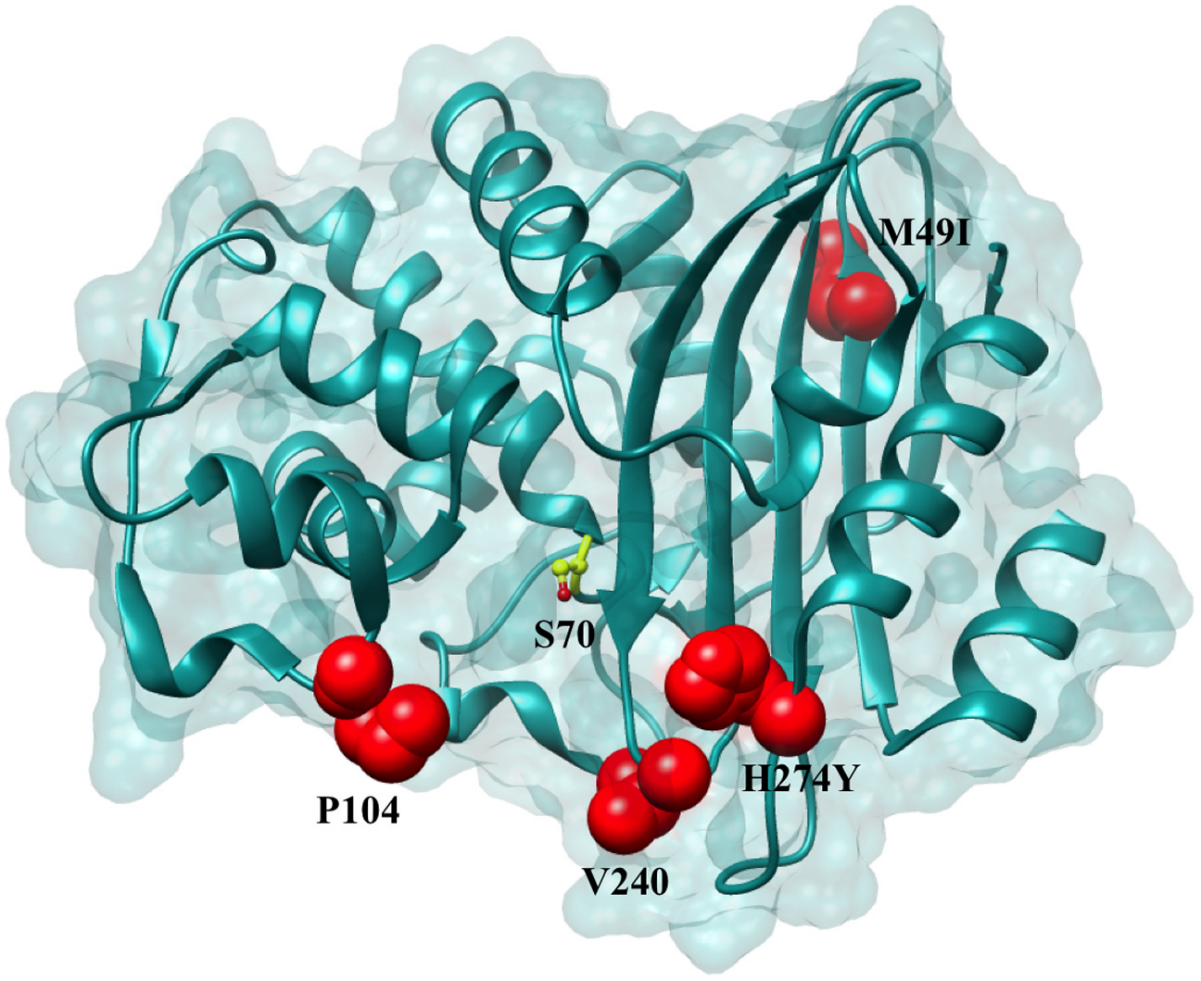
Positions of the variants residues included in this study on the KPC-2 enzyme. Positions that are substituted in variant enzymes are highlighted in red. The catalytic Ser70 is represented in yellow as a ball and stick model.

Biochemical data have shown that KPC-2 is effective in hydrolyzing penicillins, cephalosporins and carbapenems. However, KPC-2 hydrolyzes cephamycins and ceftazidime poorly [16]. This broad-spectrum activity has resulted in severely limited treatment options leading to high fatality rates [14]. The problem of antibiotic resistance due to KPC enzymes has been compounded by the recent identification of a number of clinical variants of KPC-2. Currently, a total of 22 KPC variants have been annotated by Genbank and listed on the Lahey Clinic website (http://www.lahey.org/studies/other.asp#table1). In this study, we have characterized the variants KPC-3 to KPC-11 that possess 1 to 2 amino acid substitutions as compared to KPC-2. KPC-2 was first isolated in North Carolina, however the KPC variants have been isolated from Columbia, Italy, Spain, Puerto Rico, Scotland and Israel, indicating that this enzyme has rapidly disseminated throughout the world [17–22] (Fig 2). While KPC-2 was named after *Klebsiella pneumoniae*, these variants have been isolated from a variety of organisms including *E*. *coli*, *Enterobacter cloacae*, *Actinobacter calcoaceticus-baumannii* and *Pseudomonas aeroginosa*. [17–22] (Table 1).

**Table 1:** Nucleotide polymorphisms and amino acid changes in variants as compared to KPC-2.

Variant	Nucleotide Change	Amino Acid Change	GenBank ID
KPC-3	814 C --> T	H274Y	AF395881
KPC-4	308 C --> G	P104R	AY700571
	716 T --> G	V240G	
KPC-5	308 C --> G	P104R	EU400222
KPC-6	716 T --> G	V240G	EU555534
KPC-7	147 G --> A	M49I	EU729727
	814 C --> T	H274Y	
KPC-8	716 T --> G	V240G	FJ234412
	814 C --> T	H274Y	
KPC-9	716 T --> C	V240A	FJ624872
	814 C --> T	H274Y	
KPC-10	308 C --> G	P104R	GQ140348
	814 C --> T	H274Y	
KPC-11	308 C --> T	P104L	HM066995

Previous studies on KPC 3–6 and 9–11 have indicated that the amino acid substitutions associated with these variants result in increased resistance to ceftazidime while not affecting carbapenem resistance [17–22]. However, there is a need for more detailed biochemical analysis of the effect of these substitutions on the enzyme kinetics, stability and substrate profile of KPC-2. Additionally, no information about the substrate profiles is available for the variants KPC-7 and KPC-8. This information is essential to determine treatment regimens and for the design of new inhibitors for these enzymes. The results presented here indicate that the acquired mutations in KPC-2 expand its substrate profile by increasing catalytic efficiency for ceftazidime hydrolysis as much as 80-fold compared to wild-type KPC-2. This study also highlights the evolutionary advantage conferred to KPC-2 as compared to other class A β-lactamases due to its high structural stability and consequent ability to acquire destabilizing mutations while still maintaining a folded, active structure.

## Results

### Resistance profiles of KPC-2 variants expressed in *E*. *coli*

A total of nine KPC-2 variants, four variants differing from KPC-2 by a single amino acid and five variants differing from KPC-2 by two amino acids, were constructed by site-directed mutagenesis and introduced into *E*. *coli* RB791 cells (Materials and Methods). The same strain containing the expression plasmid that did not encode KPC-2 was used as a negative control. The effect of the mutations on the resistance profiles in *E*. *coli* was evaluated by determining MIC’s for each variant for a penicillin (ampicillin), an oxyimino-cephalosporin (ceftazidime) and the carbapenems, imipenem and meropenem (Table 2). Comparing the resistance profiles of the variant enzymes in an identical genetic background allows assignment of any changes in resistance to the corresponding single or double amino acid change, thus highlighting the role of specific residues in resistance to specific substrates (Table 2).

**Table 2:** Minimum inhibitory concentrations (MIC’s) of antibiotics for KPC variants.

		MIC (μg / mL)			
		AMP	CAZ	IMI	MERO
pTP123-*empty*		16	0.125	0.38	0.064
pTP123-*bla*_KPC-2_	(KPC-2)	128	0.38	1	0.38
P104R	(KPC-5)	64	2.0	0.75	0.25
P104L	(KPC-11)	64	0.5	0.75	0.25
V240G	(KPC-6)	128	1.5	1.0	0.25
H274Y	(KPC-3)	64	1.5	1	0.25
P104R:V240G	(KPC-4)	64	12	1	0.25
P104R:H274Y	(KPC-10)	32	16	1	0.125
V240A:H274Y	(KPC-9)	128	4	1	0.19
V240G:H274Y	(KPC-8)	128	32	2	0.25
M49I:H274Y	(KPC-7)	64	1.5	1	0.25

The MIC values of the four single amino acid variants, H274Y (KPC-3), P104R (KPC-5), V240G (KPC-6) and P104L (KPC-11) for ampicillin, imipenem and meropenem were within 2-fold of the KPC-2 MIC’s for these substrates. Interestingly, the H274Y (KPC-3) and V240G (KPC-6) substitutions resulted in a 4-fold increase in resistance to ceftazidime, while the P104R (KPC-5) substitution resulted in a 5-fold increase in resistance to ceftazidime. While the P104L (KPC-11) substitution did not display any change in resistance to ceftazidime, the other clinically observed single amino acid changes in KPC-2 result in an increase in resistance to ceftazidime while maintaining the resistance levels to penicillin and carbapenem antibiotics.

Similar to the single amino acid variants, the two amino acid variants did not display any significant differences for ampicillin, imipenem and meropenem MIC’s. The only exception was P104R:H274Y (KPC-10) that displayed a 4-fold and 3-fold decrease in MIC for ampicillin and meropenem, respectively. However, consistent with the observation for the single amino acid variants, each of the double amino acid variants resulted in an increase in resistance to ceftazidime. While M49I:H274Y (KPC-7) resulted in a modest 4-fold increase in resistance to ceftazidime, V240A:H274Y (KPC-9), P104R:V240G (KPC-4), P104R:H274Y (KPC-10) and V240G:H274Y (KPC-8) resulted in 10-, 30-, 40- and 80-fold increases in ceftazidime MIC, respectively. The observation that these substitutions do not affect resistance to penicillin and carbapenem antibiotics while increasing resistance to ceftazidime implicates ceftazidime as the selective pressure for the acquisition of these variants in clinical isolates. Also, the dramatic increase in ceftazidime resistance in the two amino acid variants as compared to the single amino acid variants indicates a step-wise evolution with the acquisition increasing resistance with each amino acid variation.

### Steady-state enzyme kinetics

**Fig 3:**
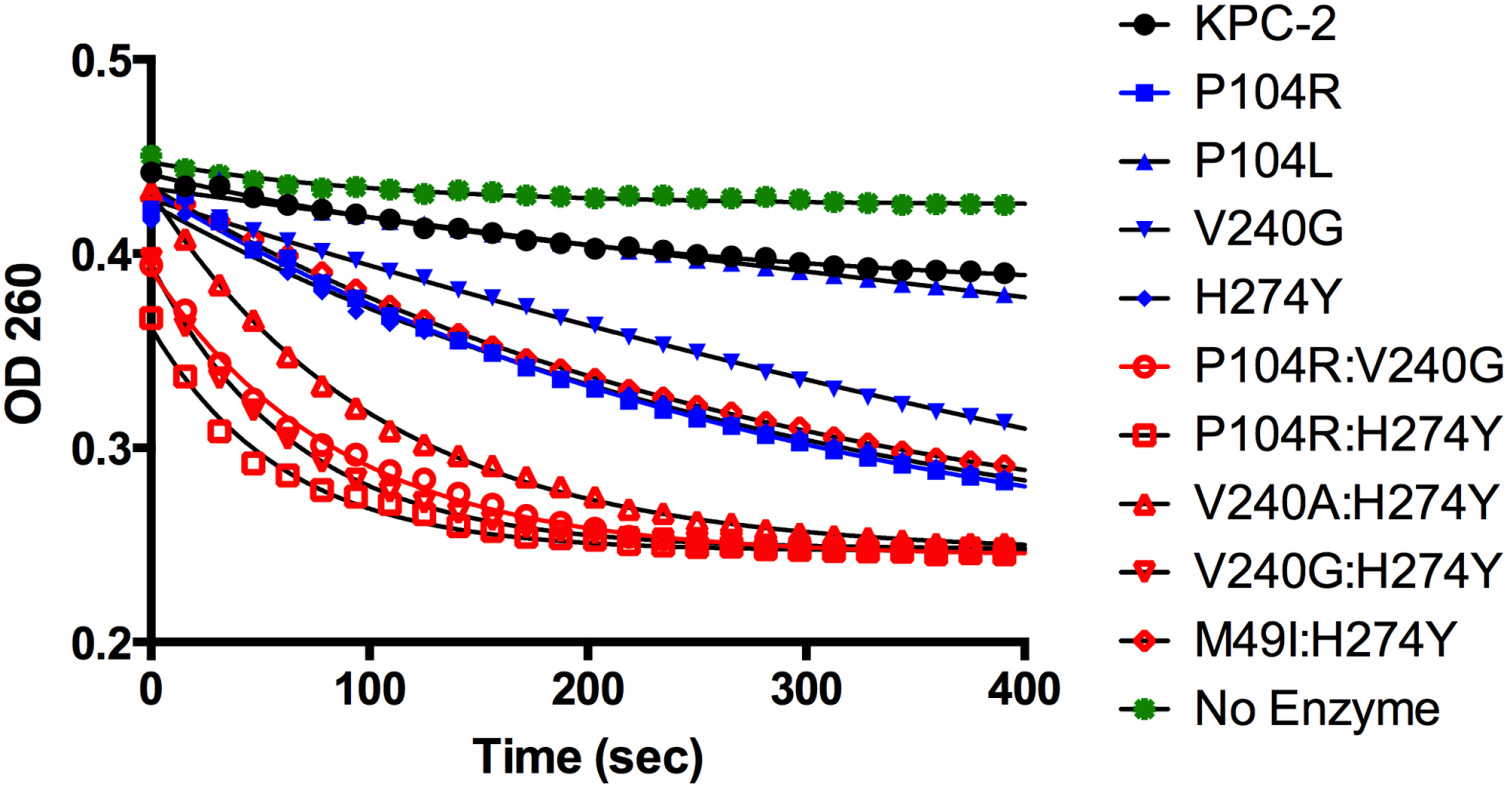
Progress curves of KPC-2 (black), single mutants (blue) and double mutants (red) and no enzyme control (green) for ceftazidime hydrolysis. All reactions were performed with 500 nM enzyme and 50 μM ceftazidime. Hydrolysis of ceftazidime results in a loss of absorbance at 260 nm.

In order to have a biochemical correlate to the MIC data, each KPC variant was purified and steady-state kinetic parameters were determined for ampicillin, imipenem, meropenem and ceftazidime (Table 3). Consistent with the MIC data, the single amino acid changes did not result in greater than 2-fold changes in catalytic efficiencies (*k*_cat_/*K*_m_) for ampicillin, imipenem and meropenem. The KPC variants, as well as the parental KPC-2, have high *K*_m_ values for ceftazidime, and saturating levels of substrate cannot be obtained. However, the *k*_cat_/*K*_m_ value was determined under conditions where [S] << *K*_m_. Although the individual *k*_cat_ and *K*_m_ values could not be determined for ceftazidime hydrolysis by KPC-2 and the variants, a progress curve of the reaction with identical amounts of enzyme and substrate clearly shows the differences in activity of the enzymes (Fig 3). The KPC-2 enzyme hydrolyzes ceftazidime poorly with a catalytic efficiency of 8 x 10^-4^ μM^-1^sec^-1^. Consistent with the ceftazidime MIC results, the P104L mutant exhibited only a modest 2-fold increase in catalytic efficiency for ceftazidime hydrolysis. In contrast, 5-fold, 9-fold and 11-fold increases were observed for the V240G, H274Y and P104R mutants, respectively. Thus, while both the P104R and P104L substitutions are found in clinical isolates, arginine at this position seems to be preferred as compared to leucine for ceftazidime hydrolysis. Both the MIC and enzymatic data suggest that mutation of Pro104 to Arg results in the highest resistance levels to ceftazidime among the single amino acid variants due to the increased ability of this variant enzyme to hydrolyze ceftazidime as compared to KPC-2.

**Table 3:** Kinetic parameters of KPC variants.

		AMP	IMI	MERO	CAZ
KPC-2	*k*_cat_ (sec^-1^)	50 ± 7	48 ± 3	3.8 ± 0.7	
	*K*_m_ (μM)	226 ± 68	252 ± 30	36 ± 16	
	*k*_cat_/K_m_ (μM^-1^.sec^-1^)	0.23 ± 0.04	0.19 ± 0.03	0.11 ± 0.036	0.0008 ± 0.0002
P104R	*k*_cat_ (sec^-1^)	314 ± 15	28 ± 1	3.0 ± 0.4	
(KPC-5)	*K*_m_ (μM)	1407 ± 123	247 ± 22	53 ± 10	
	*k*_cat_/K_m_ (μM^-1^.sec^-1^)	0.22 ± 0.01	0.12 ± 0.01	0.06 ± 0.004	0.009 ± 0.001
P104L	*k*_cat_ (sec^-1^)	12.8 ± 0.2	54 ± 4	2.5 ± 0.1	
(KPC-11)	*K*_m_ (μM)	136 ± 18	244 ± 31	35 ± 7	
	*k*_cat_/K_m_ (μM^-1^.sec^-1^)	0.1 ± 0.01	0.22 ± 0.01	0.07 ± 0.01	0.002 ± 0.0002
V240G	*k*_cat_ (sec^-1^)	146 ± 4	27 ± 1	2.3 ± 0.07	
(KPC-6)	*K*_m_ (μM)	318 ± 11	172 ± 23	30 ± 4	
	*k*_cat_/K_m_ (μM^-1^.sec^-1^)	0.46 ± 0.01	0.16 ± 0.02	0.08 ± 0.008	0.004 ± 0.0005
H274Y	*k*_cat_ (sec^-1^)	224 ± 16	34 ± 4	1.6 ± 0.1	
(KPC-3)	*K*_m_ (μM)	432 ± 48	108 ± 25	31 ± 8	
	*k*_cat_/K_m_ (μM^-1^.sec^-1^)	0.52 ± 0.02	0.32 ± 0.04	0.05 ± 0.019	0.007 ± 0.0003
P104R:V240G	*k*_cat_ (sec^-1^)	61 ± 5	21 ± 1	2.3 ± 0.04	
(KPC-4)	*K*_m_ (μM)	538 ± 84	157 ± 18	30 ± 3	
	*k*_cat_/K_m_ (μM^-1^.sec^-1^)	0.12 ± 0.01	0.14 ± 0.01	0.08 ± 0.006	0.04 ± 0.002
P104R:H274Y	*k*_cat_ (sec^-1^)	190 ± 3	31 ± 1	3.1 ± 0.07	
(KPC-10)	*K*_m_ (μM)	633 ± 42	211 ± 23	36 ± 1	
	*k*_cat_/K_m_ (μM^-1^.sec^-1^)	0.3 ± 0.02	0.15 ± 0.01	0.08 ± 0.003	0.06 ± 0.01
V240A:H274Y	*k*_cat_ (sec^-1^)	49 ± 4	27 ± 3	2.2 ± 0.2	
(KPC-9)	*K*_m_ (μM)	148 ± 24	113 ± 28	21 ± 2	
	*k*_cat_/K_m_ (μM^-1^.sec^-1^)	0.33 ± 0.03	0.24 ± 0.04	0.1 ± 0.001	0.02 ± 0.004
V240G:H274Y	*k*_cat_ (sec^-1^)	24 ± 0.3	24 ± 2	2.6 ± 0.2	
(KPC-8)	*K*_m_ (μM)	132 ± 23	110 ± 22	17 ± 2	
	*k*_cat_/K_m_ (μM^-1^.sec^-1^)	0.18 ± 0.03	0.22 ± 0.02	0.16 ± 0.007	0.03 ± 0.003
M49I:H274Y	*k*_cat_ (sec^-1^)	225 ± 26	32 ± 1	3.0 ± 0.1	
(KPC-7)	*K*_m_ (μM)	411 ± 108	120 ± 1	31 ± 3	
	*k*_cat_/K_m_ (μM^-1^.sec^-1^)	0.56 ± 0.08	0.26 ± 0.01	0.1 ± 0.006	0.006 ± 0.0002

**Fig 4:**
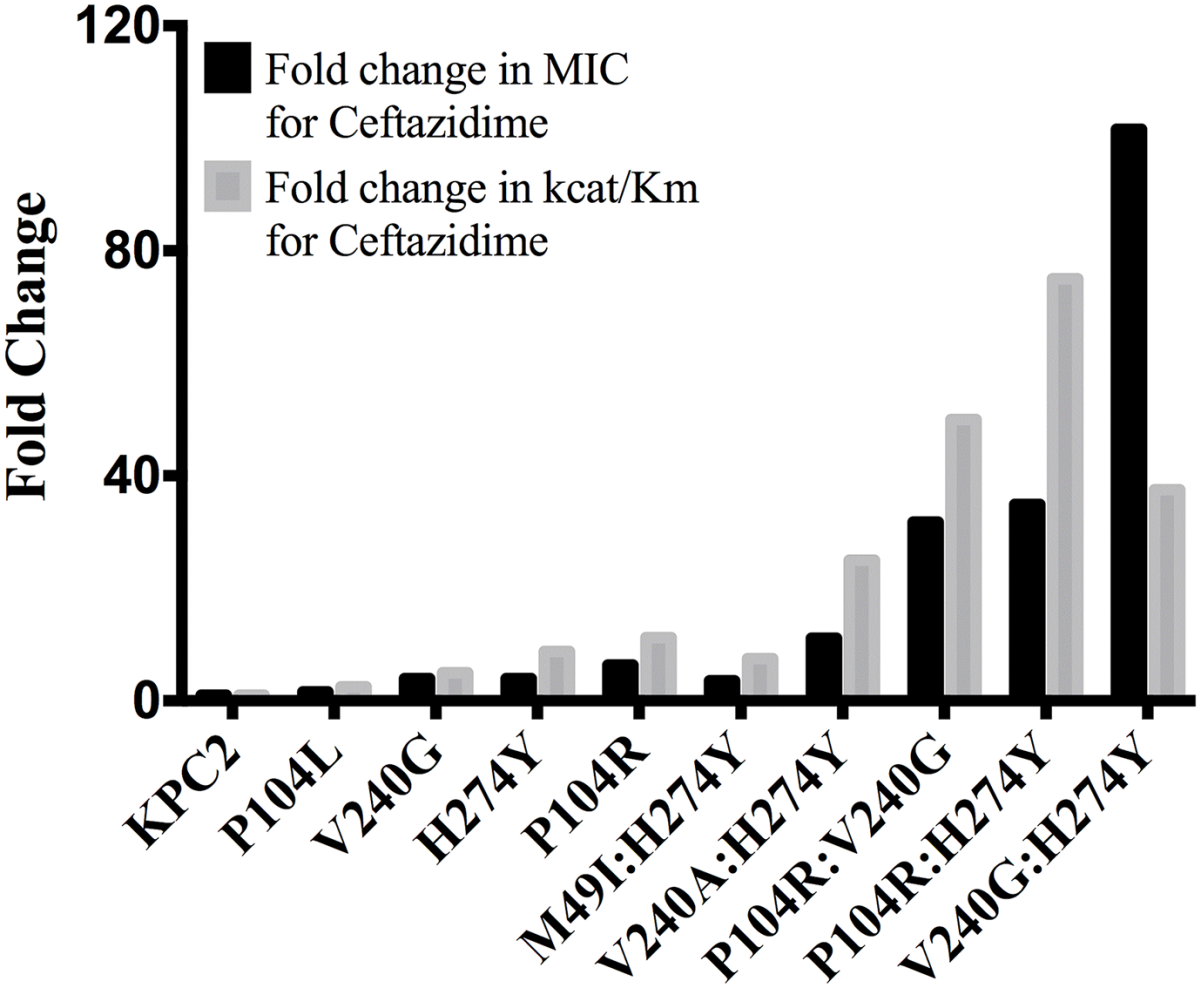
Bar graph comparing the MIC for ceftazidime (black) and catalytic efficiency for ceftazidime hydrolysis (gray). Both values are represented as fold changes compared to KPC-2.

The catalytic efficiencies of the double mutants for ampicillin, imipenem and meropenem hydrolysis remained within 2-fold of the KPC-2 catalytic efficiencies for the same substrates. Except for P104R:H274Y (KPC-10), which displayed 4-fold and 3-fold decreases in MIC for ampicillin and meropenem, respectively, the enzyme kinetic data is in agreement with the MIC values for these substrates. For ceftazidime hydrolysis, M49I:H274Y (KPC-7) had the least impact with an 8-fold increase in catalytic efficiency as compared to KPC-2. The V240A:H274Y (KPC-9) and V240G:H274Y (KPC-8) variants exhibited 25- and 40-fold increases in catalytic efficiency while the P104R:V240G (KPC-4) and P104R:H274Y (KPC-10) double mutants had the largest impact with 50- and 75-fold increases, respectively, in catalytic efficiency for ceftazidime hydrolysis (Table 3). V240G:H274Y (KPC-8) exhibited the highest MIC for ceftazidime amongst all the mutants but did not display the highest catalytic efficiency. Thus, while the V240G:H274Y (KPC-8) mutant follows the overall trend of increasing activity, its catalytic efficiency does not directly correlate with the MIC value (Fig 4). This may reflect the fact that the MIC value is influenced by a number of variables including protein expression, stability and solubility in addition to catalytic efficiency. In summary, acquisition of the single and double substitutions associated with the variants allows KPC-2 to hydrolyze ceftazidime more efficiently and broadens the substrate profiles of the enzymes (Fig 3).

### Additivity relationships between single and double amino-acid substitutions

The MIC and enzyme kinetics data indicate that the double mutants P104R:V240G (KPC-4), P104R:H274Y (KPC-10) and V240G:H274Y (KPC-8) show higher ceftazidime resistance and hydrolysis rates as compared to the constituting single mutants. When two substitutions are introduced into the enzyme together, their combined effect on catalysis may be additive or cooperative. For additive interactions, the fold change in the double mutant is expected to be the product of the fold changes for the individual mutants. However, if the two substitutions interact (directly or indirectly), the fold change in the double mutant may be much higher (or lower) than that expected from the additive effects of the two single substitutions. To determine whether the interactions between P104R, V240G and H274Y are additive or cooperative for ceftazidime hydrolysis, the free energies (ΔΔG) of the single and double mutants were calculated as described previously [23]. Briefly, the free energies associated with *k*_cat_/*K*_m_ values for KPC-2 and the single and double variants were calculated using Eq 2:

$$\Delta \Delta \text{G}=\;-\text{RT}\;\text{ln}\frac{{\left({\text{k}}_{\text{cat}}/{\text{K}}_{\text{M}}\right)}_{\text{mutant}}}{{\left({\text{k}}_{\text{cat}}/{\text{K}}_{\text{M}}\right)}_{\text{wild}-\text{type}}}$$ (2)

Subsequently, the coupling free energy (ΔG_I_) was calculated using Eq 3:

$$\Delta \Delta {\text{G}}_{\left(\text{X},\text{Y}\right)}\;=\;\Delta \Delta {\text{G}}_{\left(\text{X}\right)}+\;\Delta \Delta {\text{G}}_{\left(\text{Y}\right)}+\;\Delta {\text{G}}_{\text{I}}$$ (3)

Here ΔΔG_(x,y)_ represents the free energy difference between the wild-type and double mutant; ΔΔG_(x)_ and ΔΔG_(y)_ represent the differences in free energy between the wild-type and each single mutant, respectively, and ΔG_I_ represents the coupling free energy [24]. The KPC-2 enzyme is considered wild-type for the purposes of these comparisons. If the interactions between the single mutations are purely additive, then the coupling free energy is zero, ΔG_I_ = 0; however, if the substitutions are non-additive (positive or negative cooperativity), then ΔG_I_ ≠ 0. The resulting ΔΔG values and ΔG_I_ values are summarized in Table 4. The ΔG_I_ values for P104R:V240G (KPC-4), P104R:H274Y (KPC-10) and V240G:H274Y (KPC-8) are 0.03, 0.07 and 0.04 respectively. These values are small compared to the ΔΔG values of the individual mutants and, therefore, the P104R, V240G and H274Y residues interact additively to facilitate ceftazidime hydrolysis. This means that the individual substitutions act independently and do not influence each other’s function when present in the double mutants. It also indicates that the order in which the individual mutations that make up a double mutant occur is not important.

**Table 4:** Free energy values and additivity relationships between substituents for ceftazidime hydrolysis.

	ΔΔG	ΔGi
KPC2	0	-
P104R	-0.63	-
V240G	-0.42	-
H274Y	-0.57	-
P104R:V240G	-1.02	0.03
P104R:H274Y	-1.13	0.07
V240G:H274Y	-0.95	0.04

Calculated from *k*_cat_/K_m_ of ceftazidime hydrolysis.

### Determination of protein stability

**Fig 5:**
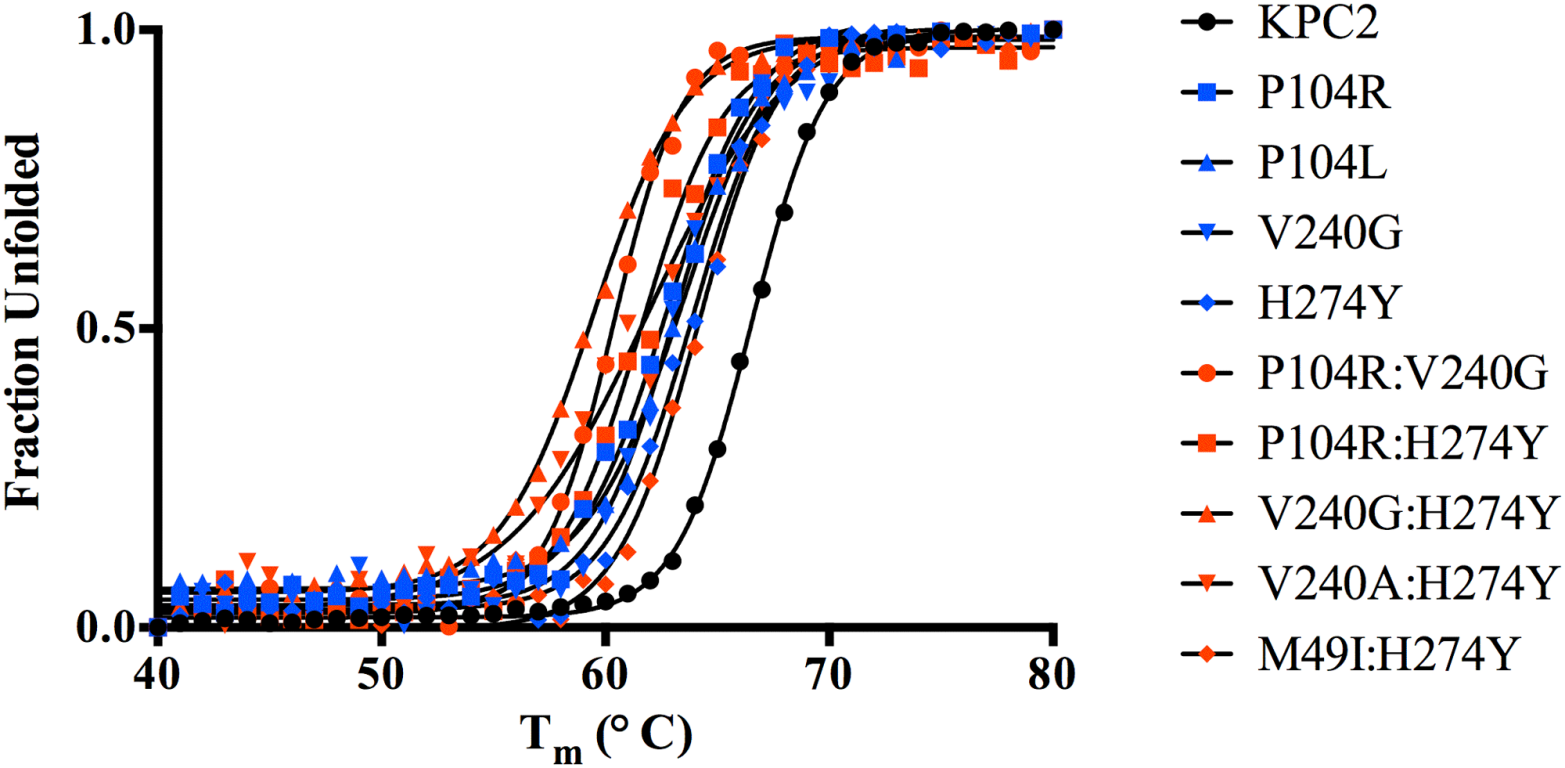
Thermal unfolding curves of KPC variants as measured by circular dichroism at 222 nm. The identity of each variant is indicated by the symbol shape and color shown in the inset.

Substitutions close to the active site that alter enzyme function are often associated with a cost in terms of loss of protein stability [25–27]. Creating new, exposed hydrophobic surfaces or polar interactions that are satisfied only when substrate binds will be destabilizing to the enzyme in the absence of substrate. In order to determine any cost associated with the substitutions in the KPC variants, the thermal stability of each purified variant enzyme was determined using circular dichroism spectroscopy by monitoring α-helix content at 222 nm with increasing temperature. The fit of the data and the *T*_m_ values of the variants are summarized in Fig 5 and Table 5. Single substitutions close to the active site result in a 2.6 to 3.7°C loss in protein stability as compared to KPC-2. With the exception of M49I:H274Y (KPC-7), the double mutants exhibited an even more dramatic reduction in *T*_m_. The P104R:V240G (KPC-4) and P104R:H274Y (KPC-10) double mutants displayed 6°C and 5°C reductions in T_m_, respectively. The V240G:H274Y (KPC-8) mutant exhibited the largest effect among all variants with a decrease in T_m_ of 7°C as compared to KPC-2. Interestingly, the V240A:H274Y (KPC-9) variant displayed a decrease in T_m_ of 5°C as compared to KPC-2. Thus, an alanine substitution at position 240 in combination with H274Y provides KPC-9 with 2°C increased stability compared to V240G:H274Y (KPC-8). Overall, the results clearly indicate that the substitutions found in the KPC variants decrease enzyme stability. Thus, the increase in ceftazidime hydrolysis resulting from the substitutions in the variants is associated with a cost in terms of stability.

**Table 5:** Melting temperatures of KPC-2 and variants.

	T_m_ (°C)	ΔTm (°C)
KPC-2	66.5	
P104R (KPC-5)	62.8	-3.7
P104L (KPC-11)	63.5	-3
V240G (KPC-6)	63	-3.5
H274Y (KPC-3)	63.9	-2.6
P104R:V240G (KPC-4)	60.4	-6.1
P104R:H274Y (KPC-10)	61.6	-4.9
V240A:H274Y (KPC-9)	61.9	-4.6
V240G:H274Y (KPC-8)	59.5	-7.0
M49I:H274Y (KPC-7)	64.1	-2.4

### Effect of mutations on protein expression levels

**Fig 6:**
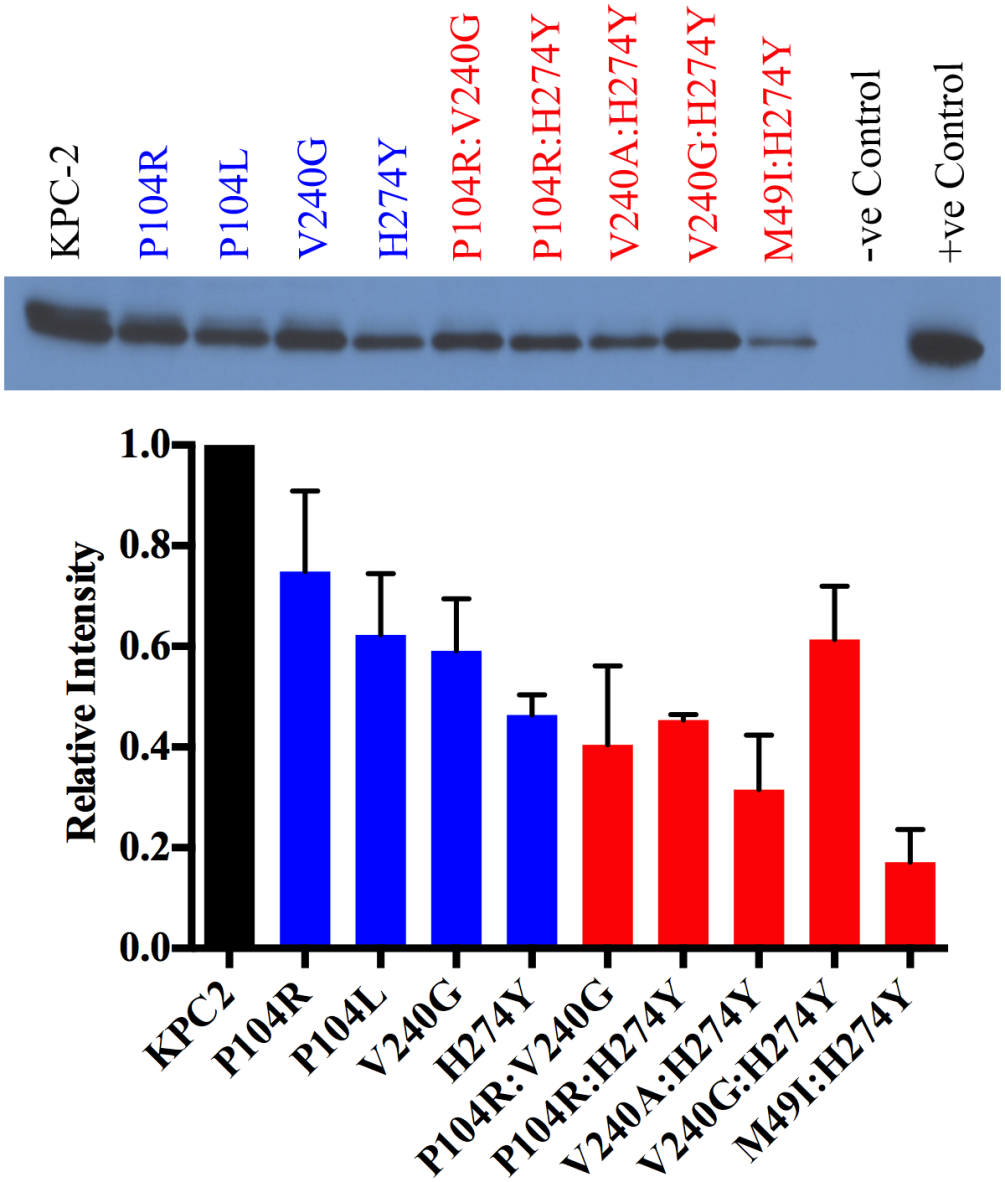
Protein expression levels of KPC-2 β-lactamase and variant enzymes. KPC-2 is represented in black, single mutants in blue and double mutants in red. Band intensities from two independent experiments were used to plot the bar graph.

In addition to thermal stability and hydrolytic activity, protein expression levels *in vivo* also contribute to the overall resistance levels. Thus, to assess the effect of the single and double mutations on protein expression and the resulting effect on resistance levels, the steady-state expression levels of KPC-2 and the variant enzymes were measured (Fig 6). As expected, KPC-2, which has the highest T_m_, also exhibits the highest expression level. The single mutants P104R, P104L and V240G showed a marginal decrease in expression while H274Y showed a 2-fold decrease. Among the double mutants, V240:H274Y and M49I:H274Y displayed the largest decrease in expression levels (3-and 4-fold respectively) while P104R:V240G and P104R:H274Y displayed a modest 2-fold decrease. The V240G:H274Y variant displayed the highest expression levels amongst all the double mutants. This provides an explanation for why this mutant showed the highest resistance to ceftazidime but not the highest catalytic efficiency (Fig 4). Taken together, the overall trends in expression levels are similar to the thermal stability results wherein the single and double mutants show a decrease in expression level as compared to KPC-2. The small magnitude of differences amongst mutants is not surprising considering that even the lowest T_m_ observed among the KPC variants is 59.5°C, which is higher as compared to other class A β-lactamases such as TEM-1 β-lactamase [28].

### In silico binding studies

**Fig 7:**
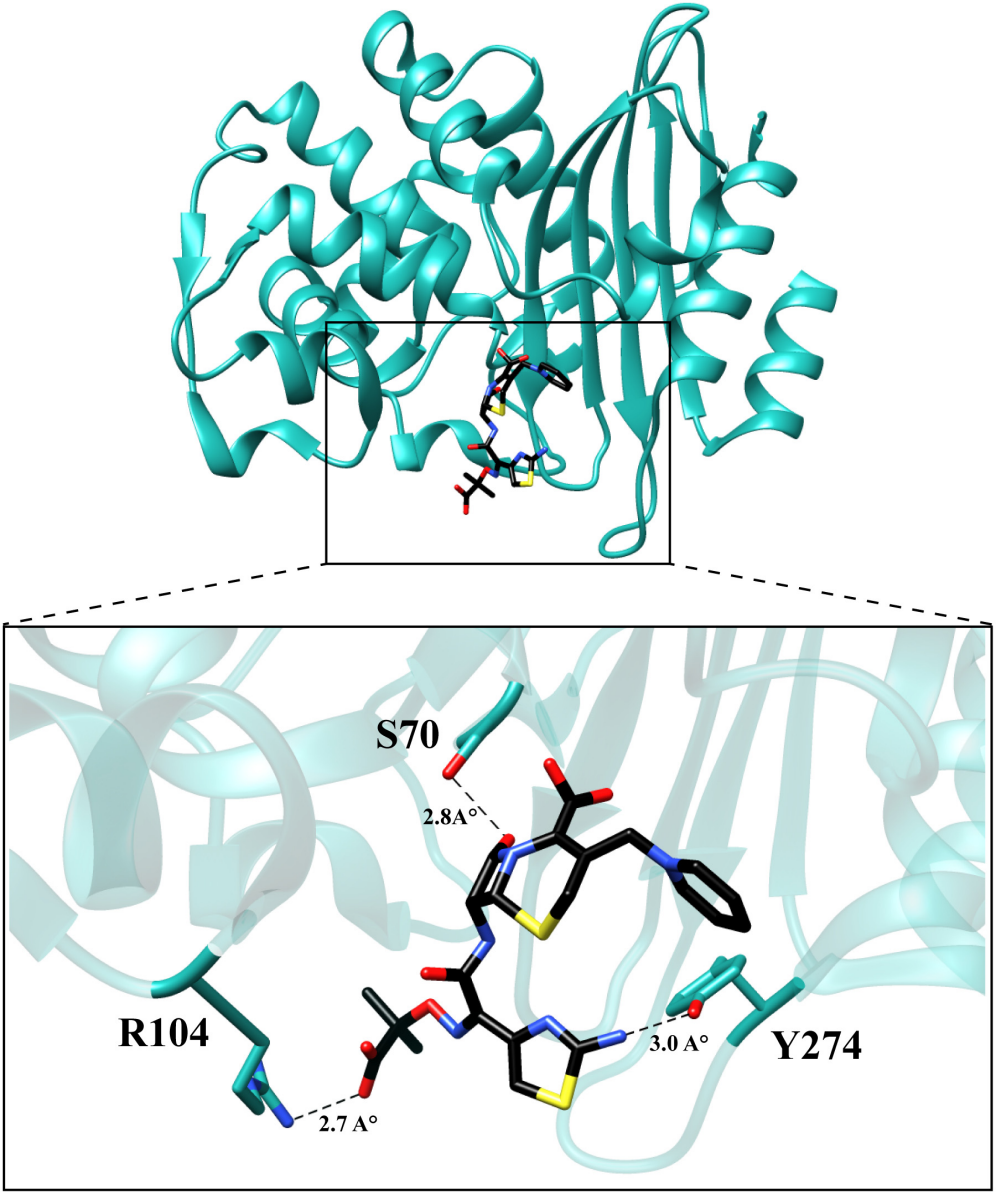
Molecular model of ceftazidime binding to the variant P104R:H274Y (KPC-10). The residues are represented in cyan and ceftazidime is represented in black. The dotted lines represent hydrogen bonds with the distances labeled.

Due to the absence of any structural data for the variants, molecular modeling was used to examine potential mechanisms by which the mutations increase the catalytic efficiencies for ceftazidime hydrolysis. Autodock Vina [29] was used to predict the binding conformation and interactions of ceftazidime with the wild-type and variant enzymes. The P104R:H274Y (KPC-10) variant was selected for study as it exhibited the largest increase in catalytic efficiency for ceftazidime hydrolysis. The KPC-2 structure was used as a starting point and the P104R and H274Y substitutions were modeled based on predicted low energy conformations (Materials and Methods) [30]. Ceftazidime was then docked into the mutant structure using Autodock Vina and the top five results were compared. The binding conformation that displayed the β-lactam carbonyl oxygen positioned in the oxyanion hole and exhibited the highest number of hydrogen bonding interactions with ceftazidime was selected for further analysis. The analysis suggests that mutating residue 104 from proline to arginine promotes hydrolysis of ceftazidime by formation of an additional hydrogen bond between the guanidinium nitrogen of the arginine and the carboxyl functionality of the oxyimino group on ceftazidime. The docking results further suggest that substitution of histidine with tyrosine at position 274 results in the formation of a hydrogen bond between the tyrosine hydroxyl side chain and the amine functionality of the aminothiazole ring. (Fig 7). These interactions could result in increased catalytic efficiency through improved substrate binding or via transition state stabilization.

## Discussion

Infections caused by bacteria producing KPC-2 β-lactamase have been associated with high rates of morbidity and mortality [14]. The presence of the KPC-2 enzyme and the consequent carbapenem-resistance reduces treatment options [14]. In recent years, a number of variants of KPC β-lactamase have been identified from patient samples covering a wide geographic distribution [17–22]. In this study, amino acid substitutions associated with several clinically isolated KPC variants were examined in an identical plasmid and genetic background to evaluate if the substitutions result in altered patterns of hydrolysis and resistance to β-lactam antibiotics. The clinically-identified single and double mutants of KPC-2 represent evolved versions of KPC-2 with an expanded substrate profile that includes the oxyimino-cephalosporin ceftazidime. Importantly, these variants do not exhibit substantially reduced activity towards carbapenems, creating a further threat to antibiotic therapy.

A broadening substrate profile through the acquisition of mutations has previously been observed for the TEM and CTX-M β-lactamases [27,31,32]. Interestingly, residue 104 plays a crucial role in conferring ceftazidime hydrolyzing ability to TEM β-lactamase [31,33]. The E104K mutation in TEM-1 results in a 4-fold increase in MIC for ceftazidime and a 50-fold increase in catalytic efficiency for ceftazidime hydrolysis [33]. Similarly, in the case of CTX-M β-lactamases, a D240G substitution increases the ceftazidime MIC by 8-fold due to a 10-fold increase in catalytic efficiency of ceftazidime hydrolysis [34]. The improved ceftazidime hydrolyzing ability of KPC-2 variants containing substitutions at residues 104 and 240 reveals that this is a common strategy among class A enzymes for expanding the substrate spectrum to ceftazidime. Interestingly, the H274Y substitution and the combinations such as P104R:V240G (KPC-4), P104R:H274Y (KPC-10), and V240G:H274Y (KPC-8) have not been associated with increased ceftazidime hydrolysis in other class A β-lactamases, suggesting these mutational pathways to ceftazidime resistance are unique to KPC-2. Modeling studies suggest that hydrogen bonds with amino acid residues at position 104 and 274 and substrate improve the catalytic efficiency of ceftazidime hydrolysis. Since glycine does not have a side-chain, modeling was not performed; however, substitution of residue 240 to glycine or alanine may expand the active site or increase flexibility in the region to accommodate ceftazidime.

**Fig 8:**
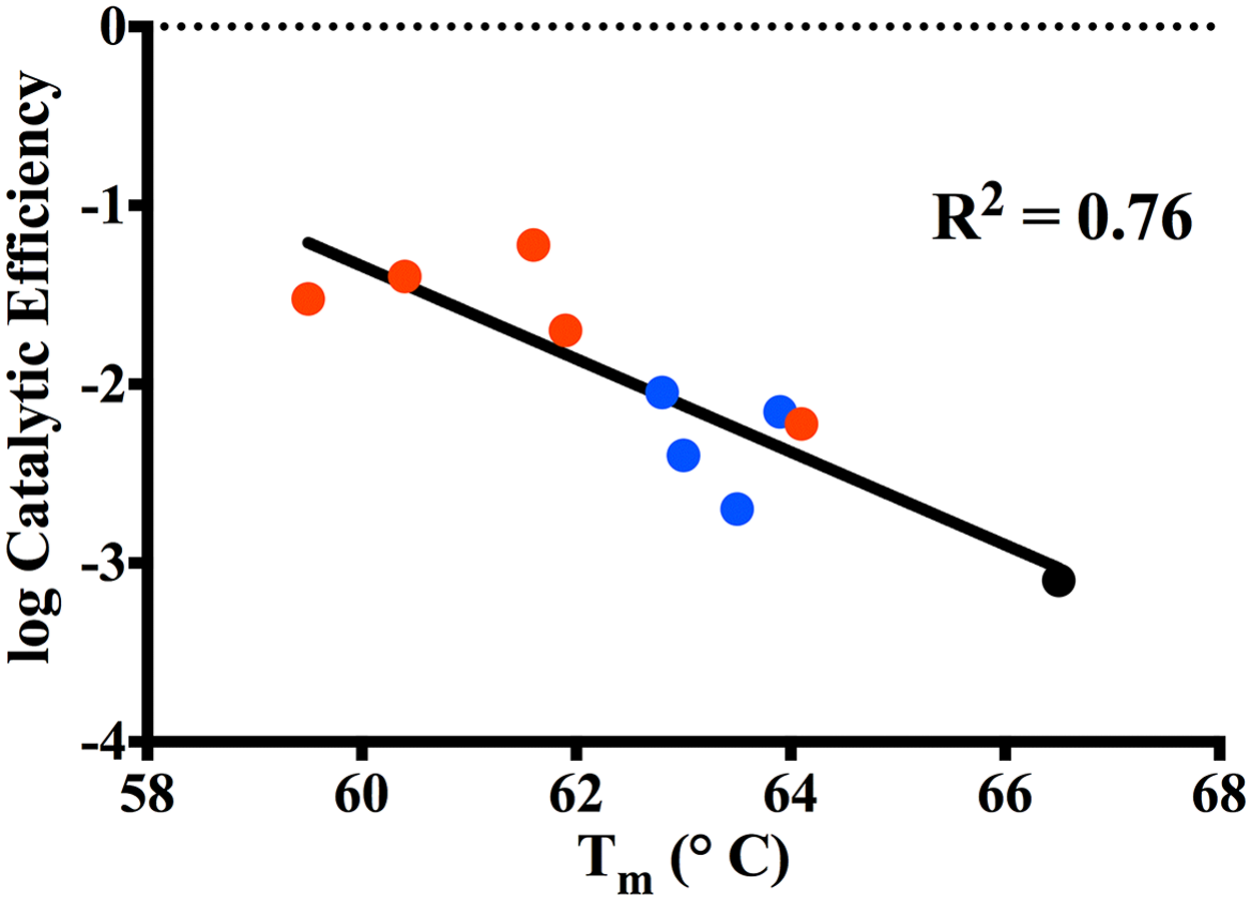
Correlation plot of log catalytic efficiency for ceftazidime (y-axis) as compared to thermal stability of the variants (x-axis). KPC-2 (black circle), Single mutants (blue circle), Double mutants (red circle).

A number of studies on class A β-lactamases as well as other enzymes indicate that mutations that alter function often lead to decreased stability [25–27,35,36]. This function-stability trade-off is attributed to the increased active site strain resulting from the increased activity associated with a gain-of-function substitution [27]. The observed decrease in stability of the single and double mutants of KPC-2 is consistent with a function-stability trade-off. This is illustrated in Fig 8 by a plot of the log of the catalytic efficiency for ceftazidime hydrolysis versus the thermal stability of the KPC mutants, which reveals a strong correlation between the gain of function and loss of stability (R^2^ = 0.8). Thus, there is a clear inverse relationship between function (catalytic efficiency) and stability for the KPC group of enzymes studied here.

The stability measurements performed in this study reveal that KPC-2 and its variants are more stable than other common class A enzymes. For example, the T_m_ of KPC-2 (66.5°C) is 15°C higher than that of TEM-1 (51.5°C) and CTX-M-14 (51°C) [28,37]. When TEM-1 acquires a single substitution such as G238S (TEM-19) that improves its oxyimino-cephalosporins hydrolyzing activity, it results in a 4.5°C decrease in T_m_ to 47°C [27]. The low stability of the TEM G238S mutant constrains the acquisition of further substitutions to those that do not significantly lower stability further or that rescue stability in the form of a global stabilizer substitution such as M182T [27,38]. The high stability of KPC-2 could serve as a buffer for the acquisition of further substitutions as reflected by the fact that the KPC-2 single mutants, although less stable than KPC-2, still retain higher stability than both wild type TEM-1 and CTX-M-14. Thus, single substitution mutants of KPC-2 have sufficient stability that they can acquire a second, functionally beneficial but destabilizing substitution and still fold into an active enzyme. In fact, even the double substitution mutants of KPC-2 retain higher stability than both the TEM-1 and CTX-M-14 enzymes. The immunoblotting experiments of KPC-2 and the variants reveals the loss in stability is associated with decreased expression levels, however, functional enzyme is still produced and provides for increased ceftazidime resistance as indicated by MIC values. Therefore, the high stability of KPC-2 may be an evolutionary advantage over other class A enzymes that allows it to acquire multiple destabilizing substitutions that increase catalysis.

The evolutionary advantage of a highly stable enzyme as a starting point for the selection of variants with altered function has been demonstrated for several systems in directed evolution experiments [39]. For example, the presence of the M182T stabilizing substitution in TEM-1 reduces the number of random single amino acid substitutions that inactivate the enzyme by one-third [40]. Similar observations have been made with a P450 enzyme as well as a thermostable chorismate mutase [41,42]. Thus, excess stability provides a buffer for an enzyme to absorb mutations that are catalytically beneficial but are associated with a stability cost, such as the KPC mutations characterized in this study.

To date 22 KPC variants have been identified. This study provides a detailed analysis of 9 variants. Besides these variants, a recent study characterized KPC-15, which exhibits increased ceftazidime activity and has the P104R, V240G, H274Y mutations as well as A120L and G147K substitutions [43]. Our results suggest that the P104R, V240G and H274Y substitutions contribute strongly to the ceftazidime hydrolysis activity of this variant. Of the remaining uncharacterized variants, KPC-13 (GenBank: HQ342889) and KPC-19 (GenBank: KJ775801) have the H274Y mutation in conjunction with D92G and N293T substitutions, respectively. Based on the presence of the H274Y mutation, we would speculate that these variants have increased ceftazidime hydrolysis activity. However, it is difficult to speculate on the roles of D92G and N293T considering their position is far away from the active site. KPC-14 (GenBank: JX524191), KPC-16 (GenBank: KC465199), KPC-17 (GenBank: KC465200) and KPC-22 (GenBank: KM379100), each possess unique mutations that have not been characterized. Additionally, while KPC-18, KPC-20 and KPC-21 have been identified and annotated a Genbank ID, their sequences are not yet available for analysis (http://www.lahey.org/studies/other.asp#table1).

As described here, the KPC enzymes have evolved substitutions that result in increased ceftazidime hydrolysis. Yet, the number of KPC variant enzymes identified to date is an order of magnitude less than the number of TEM variant extended spectrum β-lactamases (ESBLs) known (~220). One reason for this is that TEM was identified in 1963 and TEM ESBLs were first identified in 1983 while the KPC-2 was identified in the late 1990s and the first variants in 2001 [1,16,44]. Thus, there has been less time and opportunity for the evolution of KPC variants. It is also possible that the increased stability of the KPC variants relative to TEM variants results in less selective pressure for second site substitutions that stabilize KPC such as are commonly observed among TEM ESBLs; for example M182T [38]. This would lead to less diversification of the KPC enzymes versus the TEM enzymes due to the accumulation of fewer stabilizing substitutions.

The KPC enzymes represent a versatile and adaptable group. This study highlights how KPC-2 is evolving under the pressure of current antibiotic therapy towards increased resistance to the oxyimino-cephalosporin, ceftazidime. The V240G:H274Y (KPC-8) double mutant provides high-level resistance to all β-lactam classes including penicillins, cephalosporins and carbapenems antibiotics. The stability of this double mutant is 8°C lower than KPC-2, however, it is still substantially more stable than other class A enzymes. Therefore, one can expect the evolution of more KPC-variants with altered substrate profiles.

## Materials and Methods

### Bacterial strains and plasmids

*E*. *coli* K12 XL1-Blue strain (*rec*A1 *end*A1 *gyr*A96 *thi*-1 *hsd*R17 *sup*E44 *rel*A1 *lac* [F’ *proAB lacI* ^*q*^ *ZΔM15 Tn*10 (Tet^r^)] was obtained from Stratagene (La Jolla, CA) and used in site-directed mutagenesis experiments. The *E*. *coli* RB791 strain was used for protein expression, purification and MIC determinations [45]. The *bla*_KPC-2_ gene was inserted in the previously described pTP123 plasmid [46]. The resulting plasmid was used to express and purify the KPC-2 enzyme and also used as a template for site-directed mutagenesis and subsequent expression of mutant enzymes in *E*. *coli* RB791 [47].

### Site-directed mutagenesis

All KPC-2 mutants were created using the QuikChange kit (Stratagene, La Jolla, CA). Oligonucleotides were obtained from Integrated DNA Technologies (Coralville, IA). The following is the list of primers used to introduce mutations (underlined) into pTP123 KPC-2:

P104R: CAAAAATGCGCTGGTTCGCTGGTCACCCATCTC

P104L: CAAAAATGCGCTGGTTCTGTGGTCACCCATCTC

V240A: CGGAACCTGCGGAGCGTATGGCACGGCAAATG

V240G: CGGAACCTGCGGAGGGTATGGCACGGCAAATG

H274Y: CAAGGATGACAAGTACAGCGAGGCCGTCATC

M49I: CGGTGTGTACGCGATAGATACCGGCTCAG

### Minimum inhibitory concentration (MIC) determinations

Minimum inhibitory concentrations (MIC’s) for *E*. *coli* strain RB791 containing the KPC mutants was determined for imipenem, meropenem and ceftazidime using Etest strips (Ab Biodisk, Sweden) according to the manufacturers recommendations. The MIC’s of the variants for ampicillin were determined using the broth dilution method in a 100-well microtiter format. Overnight cultures of the variants were diluted into wells containing two-fold dilutions of ampicillin in a total volume of 300 μl LB broth. The plate was allowed to incubate overnight at 37°C with continuous shaking and scored for visible growth to determine the MIC.

### Protein purification

The relevant *bla*_KPC_ variant gene in plasmid pTP123 was transformed into *E*. *coli* RB791 cells and colonies were selected on LB agar containing 12.5 μg/mL chloramphenicol. A single colony was used to inoculate 20 mL LB containing 12.5 μg/mL chloramphenicol and allowed to grow overnight at 37°C. The overnight culture was added to 1 L LB broth containing 12.5 μg/mL chloramphenicol at a final dilution of 1:100 and subsequently allowed to grow to OD_600_ 0.7 at 37°C. Protein expression was induced by addition of 1 M IPTG to a final concentration of 0.2 mM and the cultures were grown at 23°C overnight. The cells were harvested by centrifugation at 4000 x *g* for 20 minutes and the pellet frozen for at least 1 hour at -80°C. To release the periplasmic contents, the pellet was resuspended in 50 mL of 10 mM Tris-HCl buffer, pH 8.0 containing 1 tablet of Complete Protease Inhibitor Cocktail (Roche Diagnostics Corporation, Indianapolis, IN) and incubated on ice for 1 hour. Subsequently, osmotic shock was initiated by addition of 50 mL of cold, sterile water. The insoluble material was pelleted by centrifugation at 10,000 *g* for 1 hour. The supernatant was filtered and passed through a HiTrap SP column (GE Healthcare, Piscataway, NJ). The P104R, P104R:V240G and P104R:H274Y mutants bound the column at pH 8.0 and were eluted using a NaCl gradient. The remaining enzyme variants were bound to the column by adjusting the buffer to pH 5.5 using MES acid and subsequently they were eluted using a NaCl gradient. The purity of the β-lactamase containing fractions was determined using SDS-PAGE and the pooled fractions were concentrated and subjected to size exclusion chromatography using a HiLoad Superdex 75 column (GE Healthcare, Piscataway, NJ). Protein concentrations were determined by measuring the optical density at 280 nm and using the following extinction coefficients for respective proteins: 39,545 M^-1^cm^-1^ was used for KPC-2, KPC-4, KPC-6, KPC-11; 41,035 M^-1^cm^-1^ for KPC-3, KPC-7, KPC-8, KPC-9, KPC-10 and 39,420 M^-1^cm^-1^ for KPC-5. All the extinction coefficients were calculated using the ‘ProtParam’ tool from the Swiss Institute of Bioinformatics online resource portal [48].

### Enzyme kinetics

Michaelis-Menten kinetic parameters for KPC-2 and the variant enzyme-substrate pairs were determined at 25°C in 50 mM sodium phosphate buffer, pH 7.0, containing 0.1 mg/mL BSA using variable amounts of enzyme depending on the enzyme-substrate pair. The initial velocities of β-lactam hydrolysis were measured on a Beckman-Coulter spectrophotometer model DU-800 (Fullerton, CA) using the following extinction coefficients: imipenem, Δε_295_ = -9000 M^-1^cm^-1^; meropenem, Δε_295_ = -10,940 M^-1^cm^-1^; ceftazidime Δε_295_ = -7600 M^-1^cm^-1^; ampicillin, Δε_235_ = -900 M^-1^cm^-1^. GraphPad Prism 5 was used to obtain the steady-state parameters by non-linear least squares fit of the data to the Michaelis-Menten equation *v = k*_cat_[S]/(*K*_m_ + [S]). The velocity of ceftazidime hydrolysis could not be saturated by measurable concentrations due to a high *K*_m_. Thus, the second order rate constant at steady-state, *k*_*cat*_ */K*_*m*_, was determined by fitting the progress curves to the equation *v = k*_cat_/*K*_m_[E][S], where [S] << *K*_m_ (eq. 1).

### Thermal denaturation

Thermal denaturation experiments were performed as described previously [28]. In short, the thermal stability of the KPC variants was measured on a Jasco J-815 circular dichroism spectropolarimeter (Jasco, Essex, UK) coupled with a Peltier effect temperature controller. A total of 0.15 mg/mL of enzyme in 50 mM sodium phosphate buffer, pH 7.0, was placed in a 0.1 cm quartz cuvette and unfolding of the proteins was observed at 222 nm by heating the samples from 40°C to 80°C in 0.1°C increments at a rate of 1°C min^-1^. The melting temperature (*T*_m_) is the temperature mid-point of protein unfolding and was determined by fitting the data to a single Boltzmann model.

### Protein expression levels

To assess the effect of the single and double mutations on KPC expression levels, a western blot was performed. Overnight cultures of RB791 cells expressing KPC-2 and the variants were diluted 1:100 into LB broth containing 12.5 μg/mL chloramphenicol. Cells were grown to OD_600_ = 0.6 at 37°C and 5 mL culture was pelleted by centrifugation at 13,000 rpm. The periplasmic contents were released by resuspending the cells in 100 μl of 10 mM Tris pH 8.0 buffer, containing 20% sucrose and osmotic shock induced by adding 100 μl of cold sterile water. The insoluble fraction was separated by centrifugation [23]. Total protein concentrations were determined using the Bradford protein assay. 1 μg of total periplasmic proteins were loaded in each well. Purified KPC-2 enzyme was used as a positive control and periplasmic fraction from cells containing the empty vector was used as a negative control. The blot was probed using polyclonal anti-KPC antibody as the primary antibody and anti-rabbit, horseradish peroxidase (HRP) conjugated antibody as the secondary antibody. SuperSignal West Pico Chemiluminescent Substrate (Thermo Scientific, Rockford, IL) was used as substrate for the secondary antibody. Band intensities were quantified using ImageJ software.

### Molecular modeling

In the absence of structural data, molecular modeling was performed to evaluate the effects of the mutations on KPC-2 β-lactamase. A molecular model of the P104R:H274Y was created by mutating the KPC-2 structure [30] (PDB ID: 2OV5) *in silico* using the Dunbrack rotamer library as a part of the UCSF Chimera software [49,50]. The Dunbrack backbone-dependent rotamer library predicts the conformation of the amino acid side-chain based on the global energy minimum of the protein. A PDB file for ceftazidime was created using the CORINA software, Molecular Networks Gmbh, Earlangen, Germany. Ceftazidime was then docked into this model to predict the Michaelis-Menten complex using the Autodock Vina docking method as described previously [29,51]. Briefly, the protein was prepared for docking by adding polar hydrogen atoms using AutoDockTools. The grid box was centered on the catalytic Ser-70 residue and the dimensions of the docking space (26 x 30 x 22 A°) were adjusted to include the entire catalytic site. Of the 5 results, the model with ceftazidime positioned in the binding conformation with the β-lactam carbonyl directed into the oxyanion hole and displaying the largest number of hydrogen bond and hydrophobic interactions was chosen for further analysis.
